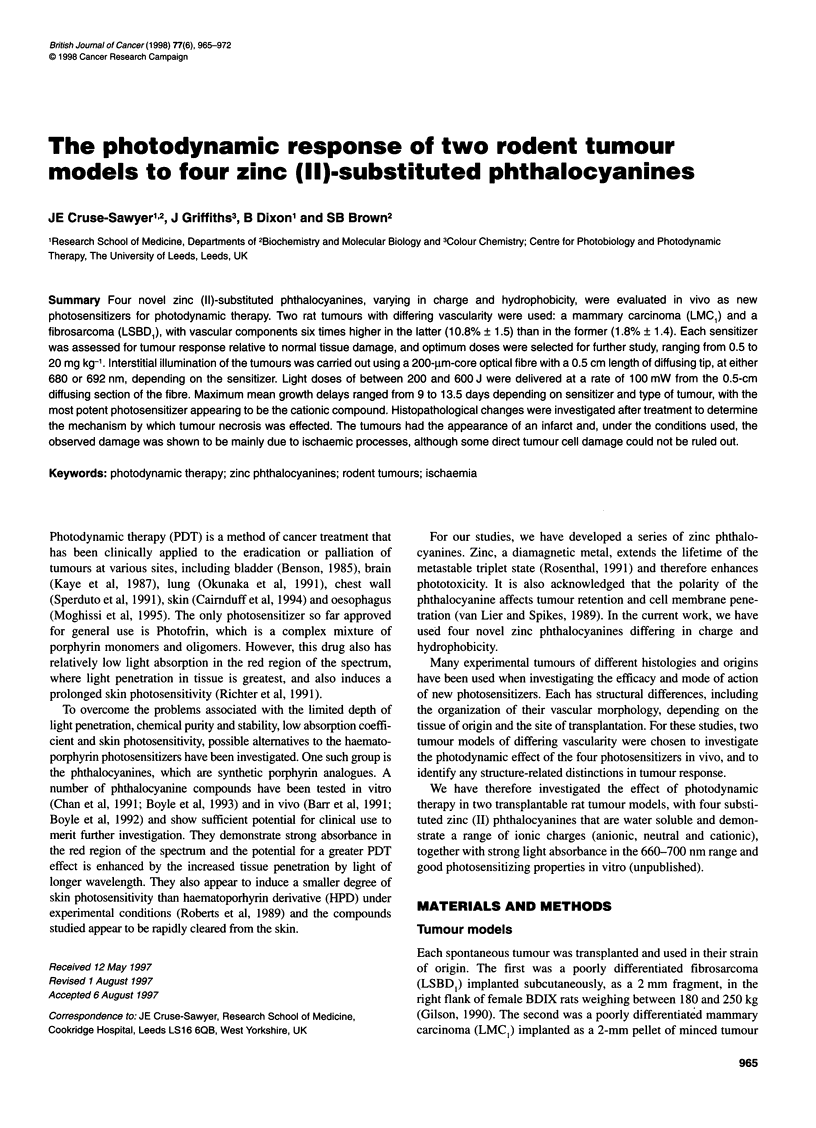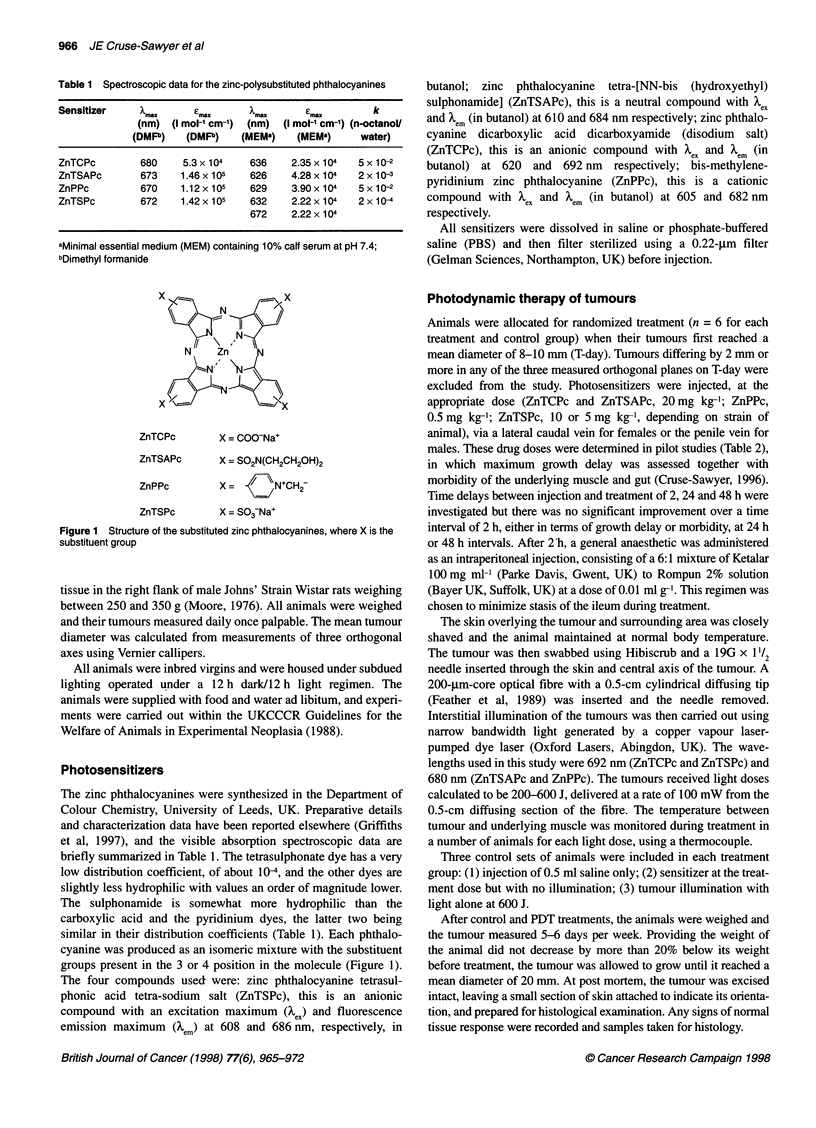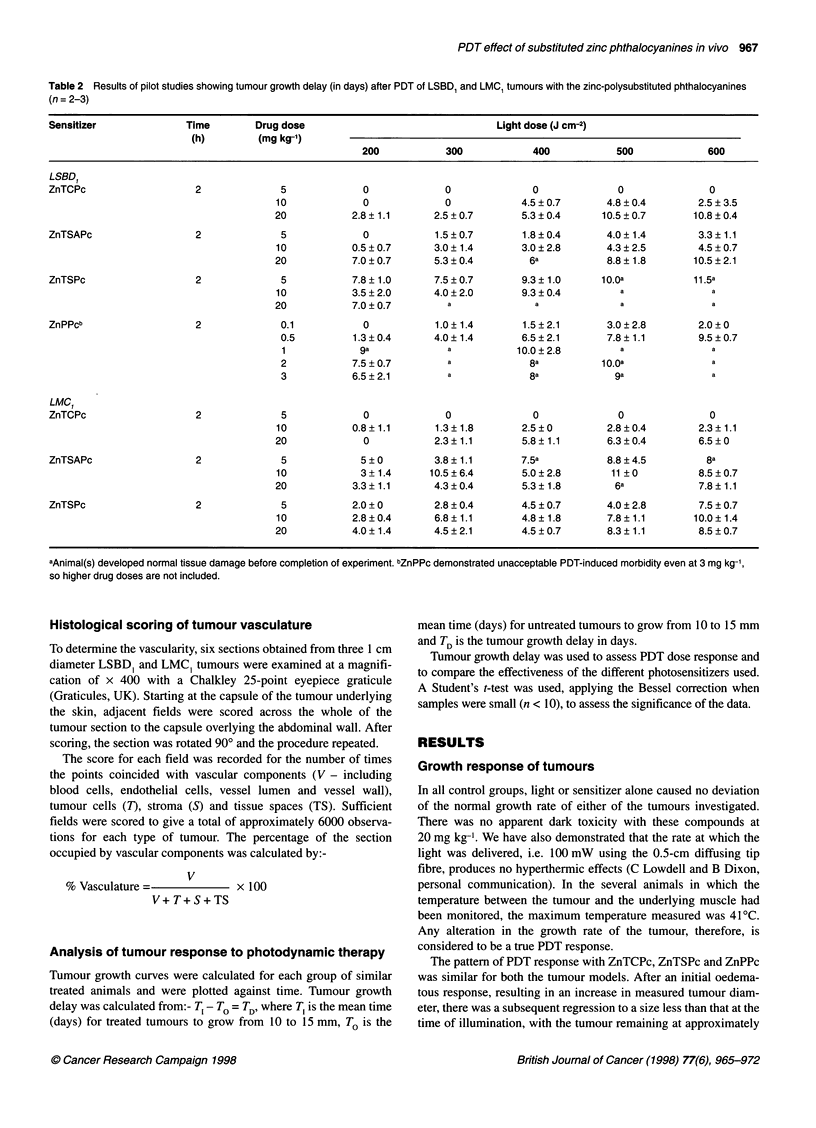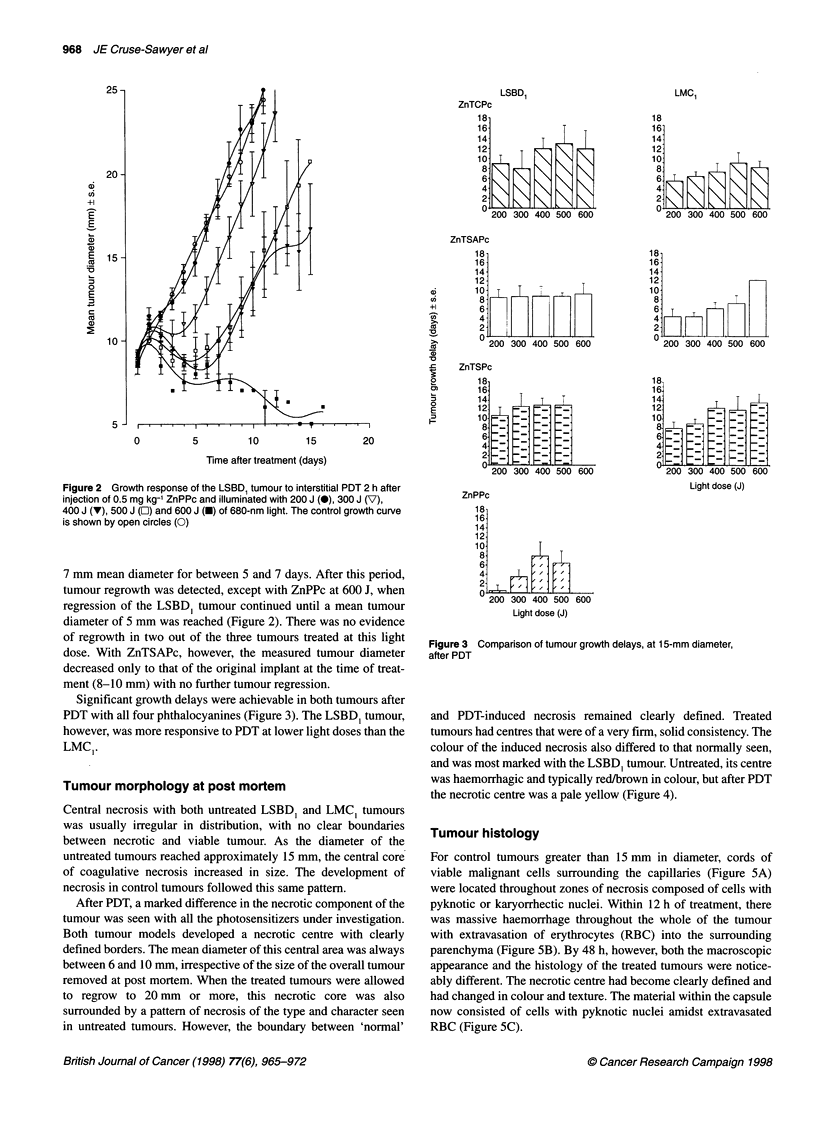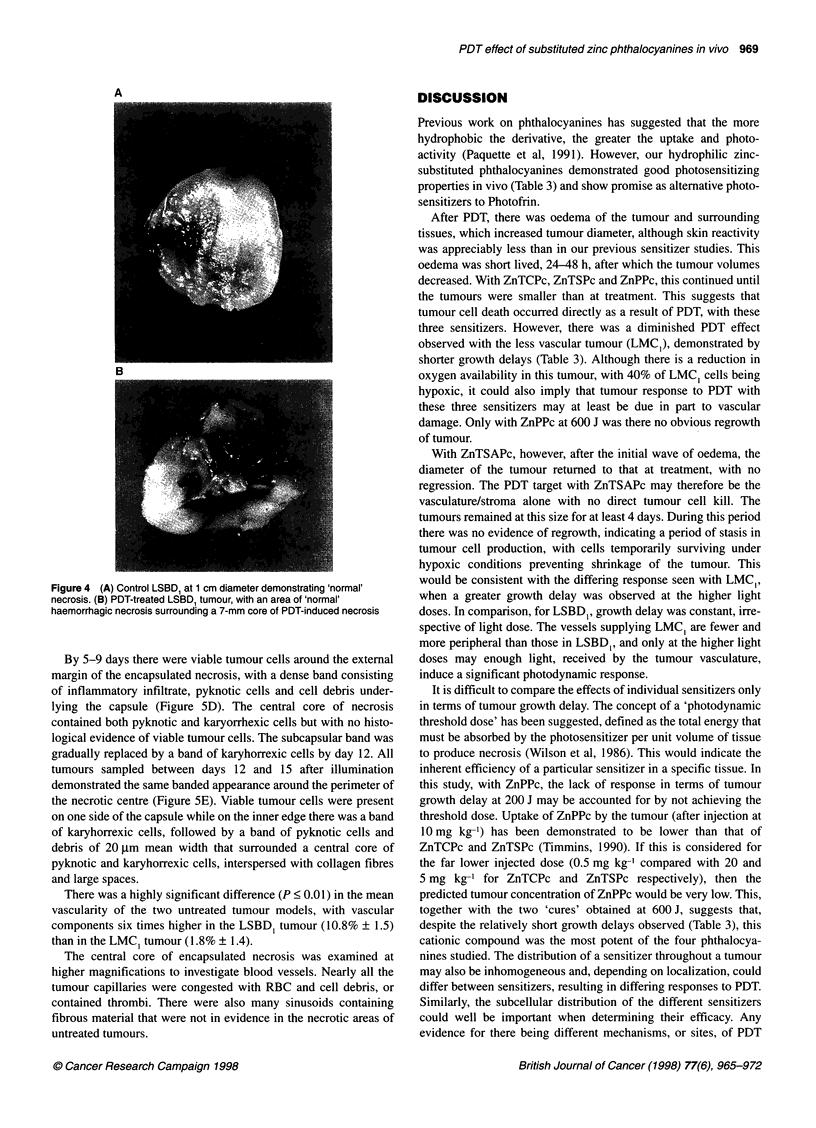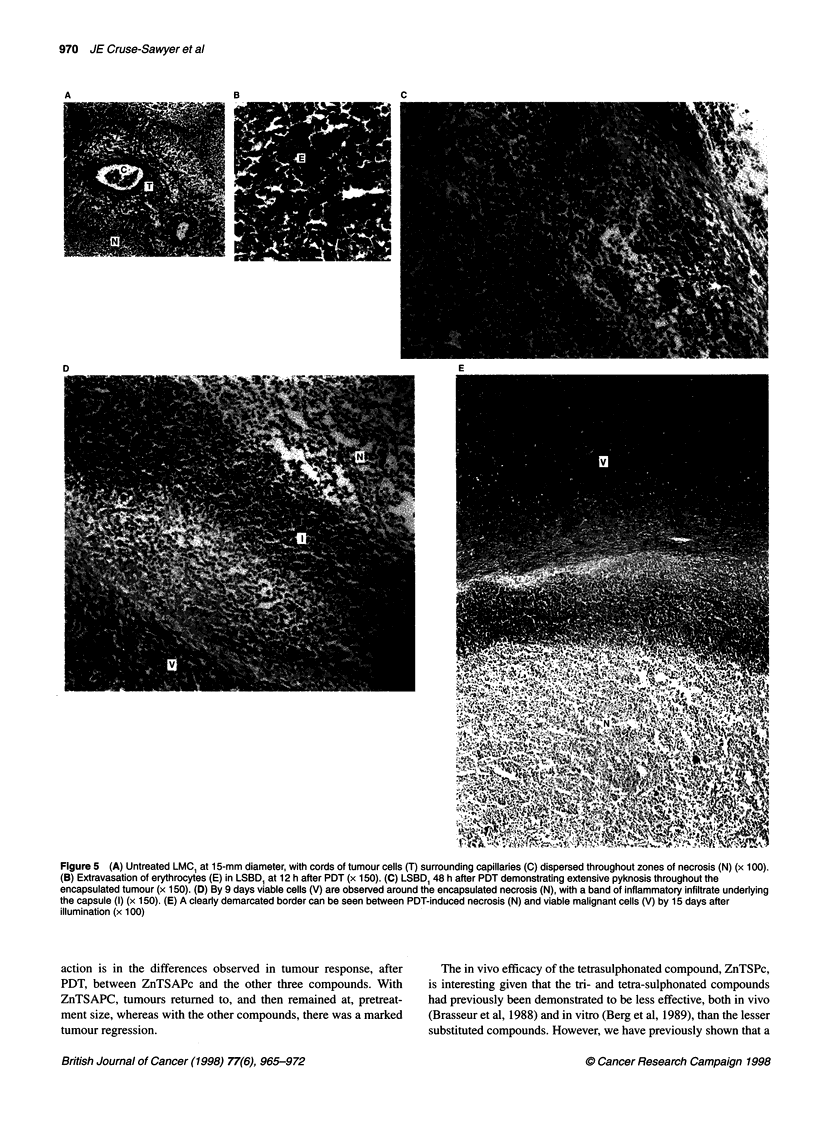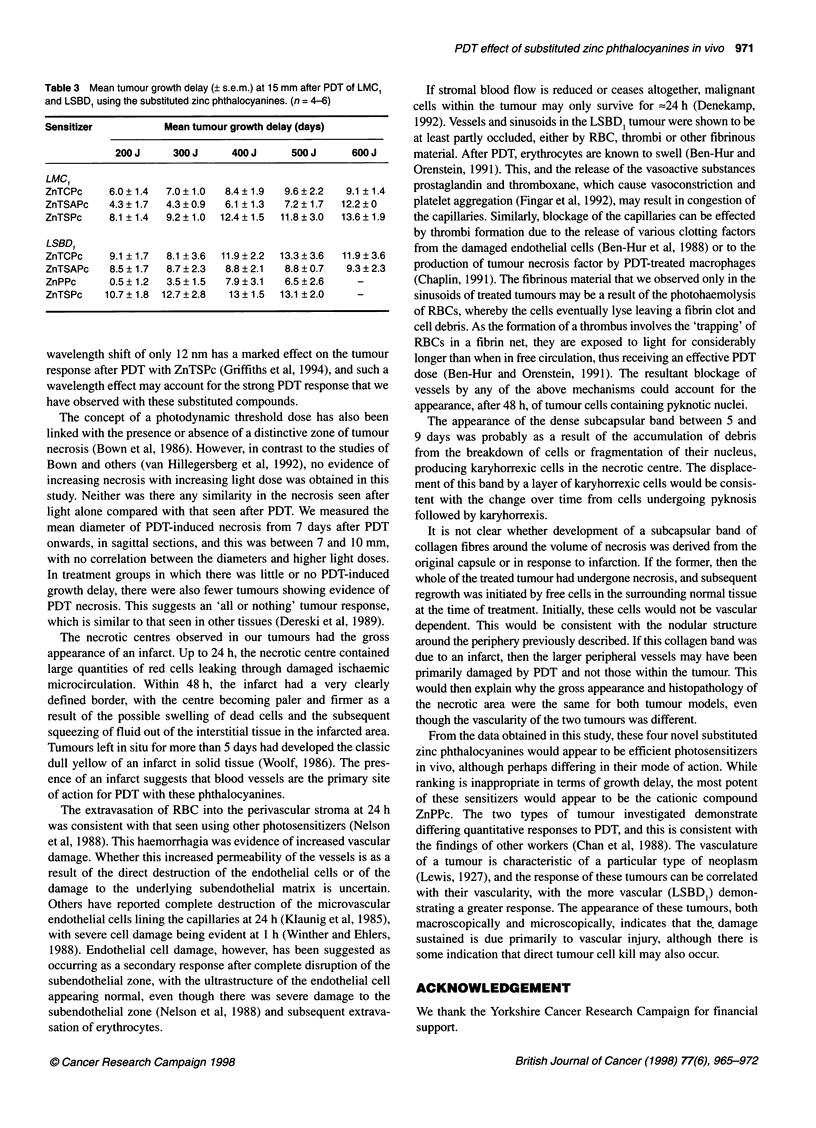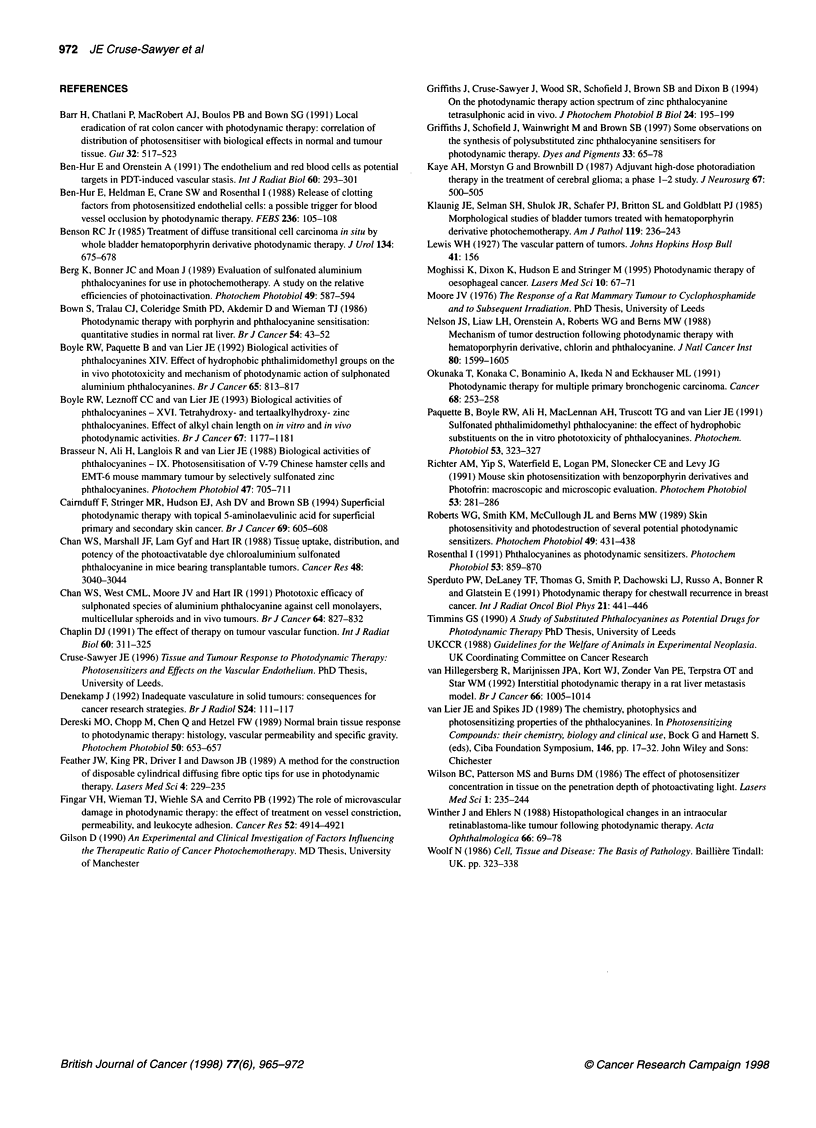# The photodynamic response of two rodent tumour models to four zinc (II)-substituted phthalocyanines.

**DOI:** 10.1038/bjc.1998.159

**Published:** 1998-03

**Authors:** J. E. Cruse-Sawyer, J. Griffiths, B. Dixon, S. B. Brown

**Affiliations:** Research School of Medicine, Department of Biochemistry and Molecular Biology, Centre for Photobiology and Photodynamic Therapy, The University of Leeds, UK.

## Abstract

**Images:**


British Journal of Cancer (1998) 77(6), 965–972
© 1998 Cancer Research Campaign

### The photodynamic response of two rodent tumour models to four zinc (II)-substituted phthalocyanines

JE Cruse-Sawyer[1,2], J Griffiths[3], B Dixon[1] and SB Brown[2]

[1]Research School of Medicine, Departments of [2]Biochemistry and Molecular Biology and [3]Colour Chemistry; Centre for Photobiology and Photodynamic Therapy, The University of Leeds, Leeds, UK

**Summary** Four novel zinc (II)-substituted phthalocyanines, varying in charge and hydrophobicity, were evaluated in vivo as new photosensitizers for photodynamic therapy. Two rat tumours with differing vascularity were used: a mammary carcinoma ($LMC_1$) and a fibrosarcoma ($LSBD_1$), with vascular components six times higher in the latter (10.8% ± 1.5) than in the former (1.8% ± 1.4). Each sensitizer was assessed for tumour response relative to normal tissue damage, and optimum doses were selected for further study, ranging from 0.5 to 20 mg $kg^{-1}$. Interstitial illumination of the tumours was carried out using a 200-µm-core optical fibre with a 0.5 cm length of diffusing tip, at either 680 or 692 nm, depending on the sensitizer. Light doses of between 200 and 600 J were delivered at a rate of 100 mW from the 0.5-cm diffusing section of the fibre. Maximum mean growth delays ranged from 9 to 13.5 days depending on sensitizer and type of tumour, with the most potent photosensitizer appearing to be the cationic compound. Histopathological changes were investigated after treatment to determine the mechanism by which tumour necrosis was effected. The tumours had the appearance of an infarct and, under the conditions used, the observed damage was shown to be mainly due to ischaemic processes, although some direct tumour cell damage could not be ruled out.

**Keywords:** photodynamic therapy; zinc phthalocyanines; rodent tumours; ischaemia

Photodynamic therapy (PDT) is a method of cancer treatment that has been clinically applied to the eradication or palliation of tumours at various sites, including bladder (Benson, 1985), brain (Kaye et al, 1987), lung (Okunaka et al, 1991), chest wall (Sperduto et al, 1991), skin (Cairnduff et al, 1994) and oesophagus (Moghissi et al, 1995). The only photosensitizer so far approved for general use is Photofrin, which is a complex mixture of porphyrin monomers and oligomers. However, this drug also has relatively low light absorption in the red region of the spectrum, where light penetration in tissue is greatest, and also induces a prolonged skin photosensitivity (Richter et al, 1991).

To overcome the problems associated with the limited depth of light penetration, chemical purity and stability, low absorption coefficient and skin photosensitivity, possible alternatives to the haematoporphyrin photosensitizers have been investigated. One such group is the phthalocyanines, which are synthetic porphyrin analogues. A number of phthalocyanine compounds have been tested in vitro (Chan et al, 1991; Boyle et al, 1993) and in vivo (Barr et al, 1991; Boyle et al, 1992) and show sufficient potential for clinical use to merit further investigation. They demonstrate strong absorbance in the red region of the spectrum and the potential for a greater PDT effect is enhanced by the increased tissue penetration by light of longer wavelength. They also appear to induce a smaller degree of skin photosensitivity than haematoporhyrin derivative (HPD) under experimental conditions (Roberts et al, 1989) and the compounds studied appear to be rapidly cleared from the skin.

*Received 12 May 1997*
*Revised 1 August 1997*
*Accepted 6 August 1997*

*Correspondence to:* JE Cruse-Sawyer, Research School of Medicine, Cookridge Hospital, Leeds LS16 6QB, West Yorkshire, UK

For our studies, we have developed a series of zinc phthalocyanines. Zinc, a diamagnetic metal, extends the lifetime of the metastable triplet state (Rosenthal, 1991) and therefore enhances phototoxicity. It is also acknowledged that the polarity of the phthalocyanine affects tumour retention and cell membrane penetration (van Lier and Spikes, 1989). In the current work, we have used four novel zinc phthalocyanines differing in charge and hydrophobicity.

Many experimental tumours of different histologies and origins have been used when investigating the efficacy and mode of action of new photosensitizers. Each has structural differences, including the organization of their vascular morphology, depending on the tissue of origin and the site of transplantation. For these studies, two tumour models of differing vascularity were chosen to investigate the photodynamic effect of the four photosensitizers in vivo, and to identify any structure-related distinctions in tumour response.

We have therefore investigated the effect of photodynamic therapy in two transplantable rat tumour models, with four substituted zinc (II) phthalocyanines that are water soluble and demonstrate a range of ionic charges (anionic, neutral and cationic), together with strong light absorbance in the 660–700 nm range and good photosensitizing properties in vitro (unpublished).

### MATERIALS AND METHODS

### Tumour models

Each spontaneous tumour was transplanted and used in their strain of origin. The first was a poorly differentiated fibrosarcoma ($LSBD_1$) implanted subcutaneously, as a 2 mm fragment, in the right flank of female BDIX rats weighing between 180 and 250 kg (Gilson, 1990). The second was a poorly differentiated mammary carcinoma ($LMC_1$) implanted as a 2-mm pellet of minced tumour

**Table 1** Spectroscopic data for the zinc-polysubstituted phthalocyanines

| Sensitizer | $\lambda_{max}$ (nm) (DMF[b]) | $\varepsilon_{max}$ ($l\ mol^{-1}\ cm^{-1}$) (DMF[b]) | $\lambda_{max}$ (nm) (MEM[a]) | $\varepsilon_{max}$ ($l\ mol^{-1}\ cm^{-1}$) (MEM[a]) | *k* (n-octanol/ water) |
|---|---|---|---|---|---|
| ZnTCPc | 680 | $5.3 \times 10^4$ | 636 | $2.35 \times 10^4$ | $5 \times 10^{-2}$ |
| ZnTSAPc | 673 | $1.46 \times 10^5$ | 626 | $4.28 \times 10^4$ | $2 \times 10^{-3}$ |
| ZnPPc | 670 | $1.12 \times 10^5$ | 629 | $3.90 \times 10^4$ | $5 \times 10^{-2}$ |
| ZnTSPc | 672 | $1.42 \times 10^5$ | 632 | $2.22 \times 10^4$ | $2 \times 10^{-4}$ |
| | | | 672 | $2.22 \times 10^4$ | |

[a]Minimal essential medium (MEM) containing 10% calf serum at pH 7.4; [b]Dimethyl formanide

**Figure 1** Structure of the substituted zinc phthalocyanines, where X is the substituent group tissue in the right flank of male Johns' Strain Wistar rats weighing between 250 and 350 g (Moore, 1976). All animals were weighed and their tumours measured daily once palpable. The mean tumour diameter was calculated from measurements of three orthogonal axes using Vernier callipers.

All animals were inbred virgins and were housed under subdued lighting operated under a 12 h dark/12 h light regimen. The animals were supplied with food and water ad libitum, and experiments were carried out within the UKCCCR Guidelines for the Welfare of Animals in Experimental Neoplasia (1988).

### Photosensitizers

The zinc phthalocyanines were synthesized in the Department of Colour Chemistry, University of Leeds, UK. Preparative details and characterization data have been reported elsewhere (Griffiths et al, 1997), and the visible absorption spectroscopic data are briefly summarized in Table 1. The tetrasulphonate dye has a very low distribution coefficient, of about $10^{-4}$, and the other dyes are slightly less hydrophilic with values an order of magnitude lower. The sulphonamide is somewhat more hydrophilic than the carboxylic acid and the pyridinium dyes, the latter two being similar in their distribution coefficients (Table 1). Each phthalocyanine was produced as an isomeric mixture with the substituent groups present in the 3 or 4 position in the molecule (Figure 1). The four compounds used were: zinc phthalocyanine tetrasulphonic acid tetra-sodium salt (ZnTSPc), this is an anionic compound with an excitation maximum ($\lambda_{ex}$) and fluorescence emission maximum ($\lambda_{em}$) at 608 and 686 nm, respectively, in butanol; zinc phthalocyanine tetra-[NN-bis (hydroxyethyl) sulphonamide] (ZnTSAPc), this is a neutral compound with $\lambda_{ex}$ and $\lambda_{em}$ (in butanol) at 610 and 684 nm respectively; zinc phthalocyanine dicarboxylic acid dicarboxyamide (disodium salt) (ZnTCPc), this is an anionic compound with $\lambda_{ex}$ and $\lambda_{em}$ (in butanol) at 620 and 692 nm respectively; bis-methylene-pyridinium zinc phthalocyanine (ZnPPc), this is a cationic compound with $\lambda_{ex}$ and $\lambda_{em}$ (in butanol) at 605 and 682 nm respectively.

All sensitizers were dissolved in saline or phosphate-buffered saline (PBS) and then filter sterilized using a 0.22-μm filter (Gelman Sciences, Northampton, UK) before injection.

### Photodynamic therapy of tumours

Animals were allocated for randomized treatment ($n = 6$ for each treatment and control group) when their tumours first reached a mean diameter of 8–10 mm (T-day). Tumours differing by 2 mm or more in any of the three measured orthogonal planes on T-day were excluded from the study. Photosensitizers were injected, at the appropriate dose (ZnTCPc and ZnTSAPc, 20 mg $kg^{-1}$; ZnPPc, 0.5 mg $kg^{-1}$; ZnTSPc, 10 or 5 mg $kg^{-1}$, depending on strain of animal), via a lateral caudal vein for females or the penile vein for males. These drug doses were determined in pilot studies (Table 2), in which maximum growth delay was assessed together with morbidity of the underlying muscle and gut (Cruse-Sawyer, 1996). Time delays between injection and treatment of 2, 24 and 48 h were investigated but there was no significant improvement over a time interval of 2 h, either in terms of growth delay or morbidity, at 24 h or 48 h intervals. After 2 h, a general anaesthetic was administered as an intraperitoneal injection, consisting of a 6:1 mixture of Ketalar 100 mg $ml^{-1}$ (Parke Davis, Gwent, UK) to Rompun 2% solution (Bayer UK, Suffolk, UK) at a dose of 0.01 ml $g^{-1}$. This regimen was chosen to minimize stasis of the ileum during treatment.

The skin overlying the tumour and surrounding area was closely shaved and the animal maintained at normal body temperature. The tumour was then swabbed using Hibiscrub and a 19G × $1^1/_2$ needle inserted through the skin and central axis of the tumour. A 200-μm-core optical fibre with a 0.5-cm cylindrical diffusing tip (Feather et al, 1989) was inserted and the needle removed. Interstitial illumination of the tumours was then carried out using narrow bandwidth light generated by a copper vapour laser-pumped dye laser (Oxford Lasers, Abingdon, UK). The wavelengths used in this study were 692 nm (ZnTCPc and ZnTSPc) and 680 nm (ZnTSAPc and ZnPPc). The tumours received light doses calculated to be 200–600 J, delivered at a rate of 100 mW from the 0.5-cm diffusing section of the fibre. The temperature between tumour and underlying muscle was monitored during treatment in a number of animals for each light dose, using a thermocouple.

Three control sets of animals were included in each treatment group: (1) injection of 0.5 ml saline only; (2) sensitizer at the treatment dose but with no illumination; (3) tumour illumination with light alone at 600 J.

After control and PDT treatments, the animals were weighed and the tumour measured 5–6 days per week. Providing the weight of the animal did not decrease by more than 20% below its weight before treatment, the tumour was allowed to grow until it reached a mean diameter of 20 mm. At post mortem, the tumour was excised intact, leaving a small section of skin attached to indicate its orientation, and prepared for histological examination. Any signs of normal tissue response were recorded and samples taken for histology.

 © Cancer Research Campaign 1998

**Table 2** Results of pilot studies showing tumour growth delay (in days) after PDT of $LSBD_1$ and $LMC_1$ tumours with the zinc-polysubstituted phthalocyanines ($n$ = 2–3)

| Sensitizer | Time (h) | Drug dose (mg kg$^{-1}$) | Light dose (J cm$^{-2}$) | | | | |
|---|---|---|---|---|---|---|---|
| | | | 200 | 300 | 400 | 500 | 600 |
| *LSBD₁* | | | | | | | |
| ZnTCPc | 2 | 5 | 0 | 0 | 0 | 0 | 0 |
| | | 10 | 0 | 0 | 4.5 ± 0.7 | 4.8 ± 0.4 | 2.5 ± 3.5 |
| | | 20 | 2.8 ± 1.1 | 2.5 ± 0.7 | 5.3 ± 0.4 | 10.5 ± 0.7 | 10.8 ± 0.4 |
| ZnTSAPc | 2 | 5 | 0 | 1.5 ± 0.7 | 1.8 ± 0.4 | 4.0 ± 1.4 | 3.3 ± 1.1 |
| | | 10 | 0.5 ± 0.7 | 3.0 ± 1.4 | 3.0 ± 2.8 | 4.3 ± 2.5 | 4.5 ± 0.7 |
| | | 20 | 7.0 ± 0.7 | 5.3 ± 0.4 | 6[a] | 8.8 ± 1.8 | 10.5 ± 2.1 |
| ZnTSPc | 2 | 5 | 7.8 ± 1.0 | 7.5 ± 0.7 | 9.3 ± 1.0 | 10.0[a] | 11.5[a] |
| | | 10 | 3.5 ± 2.0 | 4.0 ± 2.0 | 9.3 ± 0.4 | [a] | [a] |
| | | 20 | 7.0 ± 0.7 | [a] | [a] | [a] | [a] |
| ZnPPc[b] | 2 | 0.1 | 0 | 1.0 ± 1.4 | 1.5 ± 2.1 | 3.0 ± 2.8 | 2.0 ± 0 |
| | | 0.5 | 1.3 ± 0.4 | 4.0 ± 1.4 | 6.5 ± 2.1 | 7.8 ± 1.1 | 9.5 ± 0.7 |
| | | 1 | 9[a] | [a] | 10.0 ± 2.8 | [a] | [a] |
| | | 2 | 7.5 ± 0.7 | [a] | 8[a] | 10.0[a] | [a] |
| | | 3 | 6.5 ± 2.1 | [a] | 8[a] | 9[a] | [a] |
| *LMC₁* | | | | | | | |
| ZnTCPc | 2 | 5 | 0 | 0 | 0 | 0 | 0 |
| | | 10 | 0.8 ± 1.1 | 1.3 ± 1.8 | 2.5 ± 0 | 2.8 ± 0.4 | 2.3 ± 1.1 |
| | | 20 | 0 | 2.3 ± 1.1 | 5.8 ± 1.1 | 6.3 ± 0.4 | 6.5 ± 0 |
| ZnTSAPc | 2 | 5 | 5 ± 0 | 3.8 ± 1.1 | 7.5[a] | 8.8 ± 4.5 | 8[a] |
| | | 10 | 3 ± 1.4 | 10.5 ± 6.4 | 5.0 ± 2.8 | 11 ± 0 | 8.5 ± 0.7 |
| | | 20 | 3.3 ± 1.1 | 4.3 ± 0.4 | 5.3 ± 1.8 | 6[a] | 7.8 ± 1.1 |
| ZnTSPc | 2 | 5 | 2.0 ± 0 | 2.8 ± 0.4 | 4.5 ± 0.7 | 4.0 ± 2.8 | 7.5 ± 0.7 |
| | | 10 | 2.8 ± 0.4 | 6.8 ± 1.1 | 4.8 ± 1.8 | 7.8 ± 1.1 | 10.0 ± 1.4 |
| | | 20 | 4.0 ± 1.4 | 4.5 ± 2.1 | 4.5 ± 0.7 | 8.3 ± 1.1 | 8.5 ± 0.7 |

[a]Animal(s) developed normal tissue damage before completion of experiment. [b]ZnPPc demonstrated unacceptable PDT-induced morbidity even at 3 mg kg$^{-1}$, so higher drug doses are not included.

### Histological scoring of tumour vasculature

To determine the vascularity, six sections obtained from three 1 cm diameter $LSBD_1$ and $LMC_1$ tumours were examined at a magnification of × 400 with a Chalkley 25-point eyepiece graticule (Graticules, UK). Starting at the capsule of the tumour underlying the skin, adjacent fields were scored across the whole of the tumour section to the capsule overlying the abdominal wall. After scoring, the section was rotated 90° and the procedure repeated.

The score for each field was recorded for the number of times the points coincided with vascular components ($V$ – including blood cells, endothelial cells, vessel lumen and vessel wall), tumour cells ($T$), stroma ($S$) and tissue spaces (TS). Sufficient fields were scored to give a total of approximately 6000 observations for each type of tumour. The percentage of the section occupied by vascular components was calculated by:-

$$\% \text{ Vasculature} = \frac{V}{V + T + S + \text{TS}} \times 100$$

### Analysis of tumour response to photodynamic therapy

Tumour growth curves were calculated for each group of similar treated animals and were plotted against time. Tumour growth delay was calculated from:- $T_I - T_O = T_D$, where $T_I$ is the mean time (days) for treated tumours to grow from 10 to 15 mm, $T_O$ is the mean time (days) for untreated tumours to grow from 10 to 15 mm and $T_D$ is the tumour growth delay in days.

Tumour growth delay was used to assess PDT dose response and to compare the effectiveness of the different photosensitizers used. A Student's $t$-test was used, applying the Bessel correction when samples were small ($n < 10$), to assess the significance of the data.

### RESULTS

### Growth response of tumours

In all control groups, light or sensitizer alone caused no deviation of the normal growth rate of either of the tumours investigated. There was no apparent dark toxicity with these compounds at 20 mg kg$^{-1}$. We have also demonstrated that the rate at which the light was delivered, i.e. 100 mW using the 0.5-cm diffusing tip fibre, produces no hyperthermic effects (C Lowdell and B Dixon, personal communication). In the several animals in which the temperature between the tumour and the underlying muscle had been monitored, the maximum temperature measured was 41°C. Any alteration in the growth rate of the tumour, therefore, is considered to be a true PDT response.

The pattern of PDT response with ZnTCPc, ZnTSPc and ZnPPc was similar for both the tumour models. After an initial oedematous response, resulting in an increase in measured tumour diameter, there was a subsequent regression to a size less than that at the time of illumination, with the tumour remaining at approximately

© Cancer Research Campaign 1998

**Figure 2** Growth response of the $LSBD_1$ tumour to interstitial PDT 2 h after injection of 0.5 mg $kg^{-1}$ ZnPPc and illuminated with 200 J (●), 300 J (▽), 400 J (▼), 500 J (□) and 600 J (■) of 680-nm light. The control growth curve is shown by open circles (○)

**Figure 3** Comparison of tumour growth delays, at 15-mm diameter, after PDT

7 mm mean diameter for between 5 and 7 days. After this period, tumour regrowth was detected, except with ZnPPc at 600 J, when regression of the $LSBD_1$ tumour continued until a mean tumour diameter of 5 mm was reached (Figure 2). There was no evidence of regrowth in two out of the three tumours treated at this light dose. With ZnTSAPc, however, the measured tumour diameter decreased only to that of the original implant at the time of treatment (8–10 mm) with no further tumour regression.

Significant growth delays were achievable in both tumours after PDT with all four phthalocyanines (Figure 3). The $LSBD_1$ tumour, however, was more responsive to PDT at lower light doses than the $LMC_1$.

### Tumour morphology at post mortem

Central necrosis with both untreated $LSBD_1$ and $LMC_1$ tumours was usually irregular in distribution, with no clear boundaries between necrotic and viable tumour. As the diameter of the untreated tumours reached approximately 15 mm, the central core of coagulative necrosis increased in size. The development of necrosis in control tumours followed this same pattern.

After PDT, a marked difference in the necrotic component of the tumour was seen with all the photosensitizers under investigation. Both tumour models developed a necrotic centre with clearly defined borders. The mean diameter of this central area was always between 6 and 10 mm, irrespective of the size of the overall tumour removed at post mortem. When the treated tumours were allowed to regrow to 20 mm or more, this necrotic core was also surrounded by a pattern of necrosis of the type and character seen in untreated tumours. However, the boundary between 'normal' and PDT-induced necrosis remained clearly defined. Treated tumours had centres that were of a very firm, solid consistency. The colour of the induced necrosis also differed to that normally seen, and was most marked with the $LSBD_1$ tumour. Untreated, its centre was haemorrhagic and typically red/brown in colour, but after PDT the necrotic centre was a pale yellow (Figure 4).

### Tumour histology

For control tumours greater than 15 mm in diameter, cords of viable malignant cells surrounding the capillaries (Figure 5A) were located throughout zones of necrosis composed of cells with pyknotic or karyorrhectic nuclei. Within 12 h of treatment, there was massive haemorrhage throughout the whole of the tumour with extravasation of erythrocytes (RBC) into the surrounding parenchyma (Figure 5B). By 48 h, however, both the macroscopic appearance and the histology of the treated tumours were noticeably different. The necrotic centre had become clearly defined and had changed in colour and texture. The material within the capsule now consisted of cells with pyknotic nuclei amidst extravasated RBC (Figure 5C).

© Cancer Research Campaign 1998

**Figure 4** (**A**) Control $LSBD_1$ at 1 cm diameter demonstrating 'normal' necrosis. (**B**) PDT-treated $LSBD_1$ tumour, with an area of 'normal' haemorrhagic necrosis surrounding a 7-mm core of PDT-induced necrosis

By 5–9 days there were viable tumour cells around the external margin of the encapsulated necrosis, with a dense band consisting of inflammatory infiltrate, pyknotic cells and cell debris underlying the capsule (Figure 5D). The central core of necrosis contained both pyknotic and karyorrhexic cells but with no histological evidence of viable tumour cells. The subcapsular band was gradually replaced by a band of karyhorrexic cells by day 12. All tumours sampled between days 12 and 15 after illumination demonstrated the same banded appearance around the perimeter of the necrotic centre (Figure 5E). Viable tumour cells were present on one side of the capsule while on the inner edge there was a band of karyhorrexic cells, followed by a band of pyknotic cells and debris of 20 μm mean width that surrounded a central core of pyknotic and karyhorrexic cells, interspersed with collagen fibres and large spaces.

There was a highly significant difference ($P \leq 0.01$) in the mean vascularity of the two untreated tumour models, with vascular components six times higher in the $LSBD_1$ tumour (10.8% ± 1.5) than in the $LMC_1$ tumour (1.8% ± 1.4).

The central core of encapsulated necrosis was examined at higher magnifications to investigate blood vessels. Nearly all the tumour capillaries were congested with RBC and cell debris, or contained thrombi. There were also many sinusoids containing fibrous material that were not in evidence in the necrotic areas of untreated tumours.

### DISCUSSION

Previous work on phthalocyanines has suggested that the more hydrophobic the derivative, the greater the uptake and photoactivity (Paquette et al, 1991). However, our hydrophilic zinc-substituted phthalocyanines demonstrated good photosensitizing properties in vivo (Table 3) and show promise as alternative photosensitizers to Photofrin.

After PDT, there was oedema of the tumour and surrounding tissues, which increased tumour diameter, although skin reactivity was appreciably less than in our previous sensitizer studies. This oedema was short lived, 24–48 h, after which the tumour volumes decreased. With ZnTCPc, ZnTSPc and ZnPPc, this continued until the tumours were smaller than at treatment. This suggests that tumour cell death occurred directly as a result of PDT, with these three sensitizers. However, there was a diminished PDT effect observed with the less vascular tumour ($LMC_1$), demonstrated by shorter growth delays (Table 3). Although there is a reduction in oxygen availability in this tumour, with 40% of $LMC_1$ cells being hypoxic, it could also imply that tumour response to PDT with these three sensitizers may at least be due in part to vascular damage. Only with ZnPPc at 600 J was there no obvious regrowth of tumour.

With ZnTSAPc, however, after the initial wave of oedema, the diameter of the tumour returned to that at treatment, with no regression. The PDT target with ZnTSAPc may therefore be the vasculature/stroma alone with no direct tumour cell kill. The tumours remained at this size for at least 4 days. During this period there was no evidence of regrowth, indicating a period of stasis in tumour cell production, with cells temporarily surviving under hypoxic conditions preventing shrinkage of the tumour. This would be consistent with the differing response seen with $LMC_1$, when a greater growth delay was observed at the higher light doses. In comparison, for $LSBD_1$, growth delay was constant, irrespective of light dose. The vessels supplying $LMC_1$ are fewer and more peripheral than those in $LSBD_1$, and only at the higher light doses may enough light, received by the tumour vasculature, induce a significant photodynamic response.

It is difficult to compare the effects of individual sensitizers only in terms of tumour growth delay. The concept of a 'photodynamic threshold dose' has been suggested, defined as the total energy that must be absorbed by the photosensitizer per unit volume of tissue to produce necrosis (Wilson et al, 1986). This would indicate the inherent efficiency of a particular sensitizer in a specific tissue. In this study, with ZnPPc, the lack of response in terms of tumour growth delay at 200 J may be accounted for by not achieving the threshold dose. Uptake of ZnPPc by the tumour (after injection at 10 mg $kg^{-1}$) has been demonstrated to be lower than that of ZnTCPc and ZnTSPc (Timmins, 1990). If this is considered for the far lower injected dose (0.5 mg $kg^{-1}$ compared with 20 and 5 mg $kg^{-1}$ for ZnTCPc and ZnTSPc respectively), then the predicted tumour concentration of ZnPPc would be very low. This, together with the two 'cures' obtained at 600 J, suggests that, despite the relatively short growth delays observed (Table 3), this cationic compound was the most potent of the four phthalocyanines studied. The distribution of a sensitizer throughout a tumour may also be inhomogeneous and, depending on localization, could differ between sensitizers, resulting in differing responses to PDT. Similarly, the subcellular distribution of the different sensitizers could well be important when determining their efficacy. Any evidence for there being different mechanisms, or sites, of PDT

© Cancer Research Campaign 1998 

**Figure 5** (**A**) Untreated LMC$_1$ at 15-mm diameter, with cords of tumour cells (T) surrounding capillaries (C) dispersed throughout zones of necrosis (N) (× 100). (**B**) Extravasation of erythrocytes (E) in LSBD$_1$ at 12 h after PDT (× 150). (**C**) LSBD$_1$ 48 h after PDT demonstrating extensive pyknosis throughout the encapsulated tumour (× 150). (**D**) By 9 days viable cells (V) are observed around the encapsulated necrosis (N), with a band of inflammatory infiltrate underlying the capsule (I) (× 150). (**E**) A clearly demarcated border can be seen between PDT-induced necrosis (N) and viable malignant cells (V) by 15 days after illumination (× 100)

action is in the differences observed in tumour response, after PDT, between ZnTSAPc and the other three compounds. With ZnTSAPC, tumours returned to, and then remained at, pretreatment size, whereas with the other compounds, there was a marked tumour regression.

The in vivo efficacy of the tetrasulphonated compound, ZnTSPc, is interesting given that the tri- and tetra-sulphonated compounds had previously been demonstrated to be less effective, both in vivo (Brasseur et al, 1988) and in vitro (Berg et al, 1989), than the lesser substituted compounds. However, we have previously shown that a

© Cancer Research Campaign 1998

**Table 3** Mean tumour growth delay (± s.e.m.) at 15 mm after PDT of $LMC_1$ and $LSBD_1$ using the substituted zinc phthalocyanines. ($n$ = 4–6)

| Sensitizer | Mean tumour growth delay (days) | | | | |
|---|---|---|---|---|---|
| | 200 J | 300 J | 400 J | 500 J | 600 J |
| *$LMC_1$* | | | | | |
| ZnTCPc | 6.0 ± 1.4 | 7.0 ± 1.0 | 8.4 ± 1.9 | 9.6 ± 2.2 | 9.1 ± 1.4 |
| ZnTSAPc | 4.3 ± 1.7 | 4.3 ± 0.9 | 6.1 ± 1.3 | 7.2 ± 1.7 | 12.2 ± 0 |
| ZnTSPc | 8.1 ± 1.4 | 9.2 ± 1.0 | 12.4 ± 1.5 | 11.8 ± 3.0 | 13.6 ± 1.9 |
| *$LSBD_1$* | | | | | |
| ZnTCPc | 9.1 ± 1.7 | 8.1 ± 3.6 | 11.9 ± 2.2 | 13.3 ± 3.6 | 11.9 ± 3.6 |
| ZnTSAPc | 8.5 ± 1.7 | 8.7 ± 2.3 | 8.8 ± 2.1 | 8.8 ± 0.7 | 9.3 ± 2.3 |
| ZnPPc | 0.5 ± 1.2 | 3.5 ± 1.5 | 7.9 ± 3.1 | 6.5 ± 2.6 | – |
| ZnTSPc | 10.7 ± 1.8 | 12.7 ± 2.8 | 13 ± 1.5 | 13.1 ± 2.0 | – |

wavelength shift of only 12 nm has a marked effect on the tumour response after PDT with ZnTSPc (Griffiths et al, 1994), and such a wavelength effect may account for the strong PDT response that we have observed with these substituted compounds.

The concept of a photodynamic threshold dose has also been linked with the presence or absence of a distinctive zone of tumour necrosis (Bown et al, 1986). However, in contrast to the studies of Bown and others (van Hillegersberg et al, 1992), no evidence of increasing necrosis with increasing light dose was obtained in this study. Neither was there any similarity in the necrosis seen after light alone compared with that seen after PDT. We measured the mean diameter of PDT-induced necrosis from 7 days after PDT onwards, in sagittal sections, and this was between 7 and 10 mm, with no correlation between the diameters and higher light doses. In treatment groups in which there was little or no PDT-induced growth delay, there were also fewer tumours showing evidence of PDT necrosis. This suggests an 'all or nothing' tumour response, which is similar to that seen in other tissues (Dereski et al, 1989).

The necrotic centres observed in our tumours had the gross appearance of an infarct. Up to 24 h, the necrotic centre contained large quantities of red cells leaking through damaged ischaemic microcirculation. Within 48 h, the infarct had a very clearly defined border, with the centre becoming paler and firmer as a result of the possible swelling of dead cells and the subsequent squeezing of fluid out of the interstitial tissue in the infarcted area. Tumours left in situ for more than 5 days had developed the classic dull yellow of an infarct in solid tissue (Woolf, 1986). The presence of an infarct suggests that blood vessels are the primary site of action for PDT with these phthalocyanines.

The extravasation of RBC into the perivascular stroma at 24 h was consistent with that seen using other photosensitizers (Nelson et al, 1988). This haemorrhagia was evidence of increased vascular damage. Whether this increased permeability of the vessels is as a result of the direct destruction of the endothelial cells or of the damage to the underlying subendothelial matrix is uncertain. Others have reported complete destruction of the microvascular endothelial cells lining the capillaries at 24 h (Klaunig et al, 1985), with severe cell damage being evident at 1 h (Winther and Ehlers, 1988). Endothelial cell damage, however, has been suggested as occurring as a secondary response after complete disruption of the subendothelial zone, with the ultrastructure of the endothelial cell appearing normal, even though there was severe damage to the subendothelial zone (Nelson et al, 1988) and subsequent extravasation of erythrocytes.

If stromal blood flow is reduced or ceases altogether, malignant cells within the tumour may only survive for ≈24 h (Denekamp, 1992). Vessels and sinusoids in the $LSBD_1$ tumour were shown to be at least partly occluded, either by RBC, thrombi or other fibrinous material. After PDT, erythrocytes are known to swell (Ben-Hur and Orenstein, 1991). This, and the release of the vasoactive substances prostaglandin and thromboxane, which cause vasoconstriction and platelet aggregation (Fingar et al, 1992), may result in congestion of the capillaries. Similarly, blockage of the capillaries can be effected by thrombi formation due to the release of various clotting factors from the damaged endothelial cells (Ben-Hur et al, 1988) or to the production of tumour necrosis factor by PDT-treated macrophages (Chaplin, 1991). The fibrinous material that we observed only in the sinusoids of treated tumours may be a result of the photohaemolysis of RBCs, whereby the cells eventually lyse leaving a fibrin clot and cell debris. As the formation of a thrombus involves the 'trapping' of RBCs in a fibrin net, they are exposed to light for considerably longer than when in free circulation, thus receiving an effective PDT dose (Ben-Hur and Orenstein, 1991). The resultant blockage of vessels by any of the above mechanisms could account for the appearance, after 48 h, of tumour cells containing pyknotic nuclei.

The appearance of the dense subcapsular band between 5 and 9 days was probably as a result of the accumulation of debris from the breakdown of cells or fragmentation of their nucleus, producing karyhorrexic cells in the necrotic centre. The displacement of this band by a layer of karyhorrexic cells would be consistent with the change over time from cells undergoing pyknosis followed by karyhorrexis.

It is not clear whether development of a subcapsular band of collagen fibres around the volume of necrosis was derived from the original capsule or in response to infarction. If the former, then the whole of the treated tumour had undergone necrosis, and subsequent regrowth was initiated by free cells in the surrounding normal tissue at the time of treatment. Initially, these cells would not be vascular dependent. This would be consistent with the nodular structure around the periphery previously described. If this collagen band was due to an infarct, then the larger peripheral vessels may have been primarily damaged by PDT and not those within the tumour. This would then explain why the gross appearance and histopathology of the necrotic area were the same for both tumour models, even though the vascularity of the two tumours was different.

From the data obtained in this study, these four novel substituted zinc phthalocyanines would appear to be efficient photosensitizers in vivo, although perhaps differing in their mode of action. While ranking is inappropriate in terms of growth delay, the most potent of these sensitizers would appear to be the cationic compound ZnPPc. The two types of tumour investigated demonstrate differing quantitative responses to PDT, and this is consistent with the findings of other workers (Chan et al, 1988). The vasculature of a tumour is characteristic of a particular type of neoplasm (Lewis, 1927), and the response of these tumours can be correlated with their vascularity, with the more vascular ($LSBD_1$) demonstrating a greater response. The appearance of these tumours, both macroscopically and microscopically, indicates that the damage sustained is due primarily to vascular injury, although there is some indication that direct tumour cell kill may also occur.

### ACKNOWLEDGEMENT

We thank the Yorkshire Cancer Research Campaign for financial support.

© Cancer Research Campaign 1998

### REFERENCES

Barr H, Chatlani P, MacRobert AJ, Boulos PB and Bown SG (1991) Local eradication of rat colon cancer with photodynamic therapy: correlation of distribution of photosensitiser with biological effects in normal and tumour tissue. *Gut* **32**: 517–523

Ben-Hur E and Orenstein A (1991) The endothelium and red blood cells as potential targets in PDT-induced vascular stasis. *Int J Radiat Biol* **60**: 293–301

Ben-Hur E, Heldman E, Crane SW and Rosenthal I (1988) Release of clotting factors from photosensitized endothelial cells: a possible trigger for blood vessel occlusion by photodynamic therapy. *FEBS* **236**: 105–108

Benson RC Jr (1985) Treatment of diffuse transitional cell carcinoma *in situ* by whole bladder hematoporphyrin derivative photodynamic therapy. *J Urol* **134**: 675–678

Berg K, Bonner JC and Moan J (1989) Evaluation of sulfonated aluminium phthalocyanines for use in photochemotherapy. A study on the relative efficiencies of photoinactivation. *Photochem Photobiol* **49**: 587–594

Bown S, Tralau CJ, Coleridge Smith PD, Akdemir D and Wieman TJ (1986) Photodynamic therapy with porphyrin and phthalocyanine sensitisation: quantitative studies in normal rat liver. *Br J Cancer* **54**: 43–52

Boyle RW, Paquette B and van Lier JE (1992) Biological activities of phthalocyanines XIV. Effect of hydrophobic phthalimidomethyl groups on the in vivo phototoxicity and mechanism of photodynamic action of sulphonated aluminium phthalocyanines. *Br J Cancer* **65**: 813–817

Boyle RW, Leznoff CC and van Lier JE (1993) Biological activities of phthalocyanines – XVI. Tetrahydroxy- and tertaalkylhydroxy- zinc phthalocyanines. Effect of alkyl chain length on *in vitro* and *in vivo* photodynamic activities. *Br J Cancer* **67**: 1177–1181

Brasseur N, Ali H, Langlois R and van Lier JE (1988) Biological activities of phthalocyanines – IX. Photosensitisation of V-79 Chinese hamster cells and EMT-6 mouse mammary tumour by selectively sulfonated zinc phthalocyanines. *Photochem Photobiol* **47**: 705–711

Cairnduff F, Stringer MR, Hudson EJ, Ash DV and Brown SB (1994) Superficial photodynamic therapy with topical 5-aminolaevulinic acid for superficial primary and secondary skin cancer. *Br J Cancer* **69**: 605–608

Chan WS, Marshall JF, Lam Gyf and Hart IR (1988) Tissue uptake, distribution, and potency of the photoactivatable dye chloroaluminium sulfonated phthalocyanine in mice bearing transplantable tumors. *Cancer Res* **48**: 3040–3044

Chan WS, West CML, Moore JV and Hart IR (1991) Phototoxic efficacy of sulphonated species of aluminium phthalocyanine against cell monolayers, multicellular spheroids and in vivo tumours. *Br J Cancer* **64**: 827–832

Chaplin DJ (1991) The effect of therapy on tumour vascular function. *Int J Radiat Biol* **60**: 311–325

Cruse-Sawyer JE (1996) *Tissue and Tumour Response to Photodynamic Therapy: Photosensitizers and Effects on the Vascular Endothelium*. PhD Thesis, University of Leeds.

Denekamp J (1992) Inadequate vasculature in solid tumours: consequences for cancer research strategies. *Br J Radiol* **S24**: 111–117

Dereski MO, Chopp M, Chen Q and Hetzel FW (1989) Normal brain tissue response to photodynamic therapy: histology, vascular permeability and specific gravity. *Photochem Photobiol* **50**: 653–657

Feather JW, King PR, Driver I and Dawson JB (1989) A method for the construction of disposable cylindrical diffusing fibre optic tips for use in photodynamic therapy. *Lasers Med Sci* **4**: 229–235

Fingar VH, Wieman TJ, Wiehle SA and Cerrito PB (1992) The role of microvascular damage in photodynamic therapy: the effect of treatment on vessel constriction, permeability, and leukocyte adhesion. *Cancer Res* **52**: 4914–4921

Gilson D (1990) *An Experimental and Clinical Investigation of Factors Influencing the Therapeutic Ratio of Cancer Photochemotherapy*. MD Thesis, University of Manchester

Griffiths J, Cruse-Sawyer J, Wood SR, Schofield J, Brown SB and Dixon B (1994) On the photodynamic therapy action spectrum of zinc phthalocyanine tetrasulphonic acid in vivo. *J Photochem Photobiol B Biol* **24**: 195–199

Griffiths J, Schofield J, Wainwright M and Brown SB (1997) Some observations on the synthesis of polysubstituted zinc phthalocyanine sensitisers for photodynamic therapy. *Dyes and Pigments* **33**: 65–78

Kaye AH, Morstyn G and Brownbill D (1987) Adjuvant high-dose photoradiation therapy in the treatment of cerebral glioma; a phase 1–2 study. *J Neurosurg* **67**: 500–505

Klaunig JE, Selman SH, Shulok JR, Schafer PJ, Britton SL and Goldblatt PJ (1985) Morphological studies of bladder tumors treated with hematoporphyrin derivative photochemotherapy. *Am J Pathol* **119**: 236–243

Lewis WH (1927) The vascular pattern of tumors. *Johns Hopkins Hosp Bull* **41**: 156

Moghissi K, Dixon K, Hudson E and Stringer M (1995) Photodynamic therapy of oesophageal cancer. *Lasers Med Sci* **10**: 67–71

Moore JV (1976) *The Response of a Rat Mammary Tumour to Cyclophosphamide and to Subsequent Irradiation*. PhD Thesis, University of Leeds

Nelson JS, Liaw LH, Orenstein A, Roberts WG and Berns MW (1988) Mechanism of tumor destruction following photodynamic therapy with hematoporphyrin derivative, chlorin and phthalocyanine. *J Natl Cancer Inst* **80**: 1599–1605

Okunaka T, Konaka C, Bonaminio A, Ikeda N and Eckhauser ML (1991) Photodynamic therapy for multiple primary bronchogenic carcinoma. *Cancer* **68**: 253–258

Paquette B, Boyle RW, Ali H, MacLennan AH, Truscott TG and van Lier JE (1991) Sulfonated phthalimidomethyl phthalocyanine: the effect of hydrophobic substituents on the in vitro phototoxicity of phthalocyanines. *Photochem. Photobiol* **53**, 323–327

Richter AM, Yip S, Waterfield E, Logan PM, Slonecker CE and Levy JG (1991) Mouse skin photosensitization with benzoporphyrin derivatives and Photofrin: macroscopic and microscopic evaluation. *Photochem Photobiol* **53**: 281–286

Roberts WG, Smith KM, McCullough JL and Berns MW (1989) Skin photosensitivity and photodestruction of several potential photodynamic sensitizers. *Photochem Photobiol* **49**: 431–438

Rosenthal I (1991) Phthalocyanines as photodynamic sensitizers. *Photochem Photobiol* **53**: 859–870

Sperduto PW, DeLaney TF, Thomas G, Smith P, Dachowski LJ, Russo A, Bonner R and Glatstein E (1991) Photodynamic therapy for chestwall recurrence in breast cancer. *Int J Radiat Oncol Biol Phys* **21**: 441–446

Timmins GS (1990) *A Study of Substituted Phthalocyanines as Potential Drugs for Photodynamic Therapy* PhD Thesis, University of Leeds

UKCCR (1988) *Guidelines for the Welfare of Animals in Experimental Neoplasia*. UK Coordinating Committee on Cancer Research van Hillegersberg R, Marijnissen JPA, Kort WJ, Zonder Van PE, Terpstra OT and Star WM (1992) Interstitial photodynamic therapy in a rat liver metastasis model. *Br J Cancer* **66**: 1005–1014

van Lier JE and Spikes JD (1989) The chemistry, photophysics and photosensitizing properties of the phthalocyanines. In *Photosensitizing Compounds: their chemistry, biology and clinical use*, Bock G and Harnett S. (eds), Ciba Foundation Symposium, **146**, pp. 17–32. John Wiley and Sons: Chichester

Wilson BC, Patterson MS and Burns DM (1986) The effect of photosensitizer concentration in tissue on the penetration depth of photoactivating light. *Lasers Med Sci* **1**: 235–244

Winther J and Ehlers N (1988) Histopathological changes in an intraocular retinablastoma-like tumour following photodynamic therapy. *Acta Ophthalmologica* **66**: 69–78

Woolf N (1986) *Cell, Tissue and Disease: The Basis of Pathology*. Baillière Tindall: UK. pp. 323–338

© Cancer Research Campaign 1998